# 5-Hydroxymethylcytosine correlates with epigenetic regulatory mutations, but may not have prognostic value in predicting survival in normal karyotype acute myeloid leukemia

**DOI:** 10.18632/oncotarget.14171

**Published:** 2016-12-26

**Authors:** Jae-Sook Ahn, Hyeoung-Joon Kim, Yeo-Kyeoung Kim, Seung-Shin Lee, Seo-Yeon Ahn, Sung-Hoon Jung, Deok-Hwan Yang, Je-Jung Lee, Hee Jeong Park, Seung Hyun Choi, Chul Won Jung, Jun-Ho Jang, Hee Je Kim, Joon Ho Moon, Sang Kyun Sohn, Jong-Ho Won, Sung-Hyun Kim, Szardenings Michael, Mark D. Minden, Dennis Dong Kim Hwan

**Affiliations:** ^1^ Hematology-Oncology, Chonnam National University Hwasun Hospital, Jeollanam-do, Republic of Korea; ^2^ Genomic Research Center for Hematopoietic Diseases, Chonnam National University Hwasun Hospital, Jeollanam-do, Republic of Korea; ^3^ Division of Hematology-Oncology, Samsung Medical Center, Seoul, Republic of Korea; ^4^ Department of Hematology, Cancer Research Institute, Seoul St. Mary's Hospital, College of Medicine, The Catholic University of Korea, Seoul, Republic of Korea; ^5^ Department of Hematology-Oncology, Kyungpook National University Hospital, Daegu, Republic of Korea; ^6^ Department of Hematology-Oncology, Soon Chun Hyang University Hospital, Seoul, Republic of Korea; ^7^ Department of Hematology-Oncology, Dong-A University *College* of Medicine, Busan, Republic of Korea; ^8^ Fraunhofer Institute for Cell Therapy and Immunology, Leipzig, Germany; ^9^ Department of Medical Oncology and Hematology, Princess Margaret Cancer Centre, University of Toronto, Toronto, Canada

**Keywords:** TET2, IDH1/2, 5hmC, normal karyotype, AML

## Abstract

Stem cells display remarkably high levels of 5-hydroxymethylcytosine (5hmC). Both *TET2* and *IDH1/2* mutations can impair the production of 5hmC, thus decreasing 5hmC levels. *TET2* or *IDH1/2* mutations are commonly observed in acute myeloid leukemia (AML). However, the implications of 5hmC on survival in normal karyotype AML patients have not been fully evaluated. The 5hmC levels were analyzed in 375 patients using ELISA. The levels of 5hmC in DNA samples were converted to a log scale for the analysis and correlations with *TET2* and/or *IDH1/2* mutations were evaluated. The median 5hmC level was 0.065% (range 0.001–0.999). Mutation rates were 13.1% for *TET2*^mut^, 6.7% for *IDH1*^mut^, and 13.9% for *IDH2*^mut^. The prevalence of *TET2* and/or IDH1/2 was 33.1% (124/375). *TET2* and *IDH1*/*2* mutated patients had significantly lower levels of log(5hmC) compared with patients without *TET2* or *IDH1/2* mutations (*p*<0.001). With a median follow-up of 55.5 months (range, 0.7–179.8), there was no significant difference in overall survival, event-free survival, and relapse risk according to *TET2*^mut^ or *IDH1/2*^mut^ (all, *p*>0.05). To identify its prognostic value, we sub-classified the levels of 5hmC into tertiles for 5hmC values. However, there was no significant association between the categories of 5hmC levels and survival or relapse risk (all *p*>0.05). Patients with *TET2* or *IDH1/2* mutations had lower levels of 5hmC. The 5hmC levels may not be predictive of survival in patients with normal karyotype AML.

## INTRODUCTION

DNA methylation regulates the expression of specific genes and therefore plays a critical role in development, contributing to normal cellular differentiation, genomic stability, X-chromosome inactivation, and genomic imprinting [1]. The balance between methylation and demethylation is controlled by several proteins and cofactors. This balance is frequently deregulated in cancer, leading to aberrant methylation patterns [2]. The regulation of DNA hydroxymethylation is mediated by several factors including proteins from the *TET* family, which is responsible for the formation of 5-hydroxymethylcytosine (5hmC) [3]. *TET* proteins require α-ketoglutarate (αKG) as a co-substrate, which is produced by the family of isocitrate dehydrogenase (*IDH*) proteins [4]. *IDH* proteins catalyze the oxidative decarboxylation of isocitrate to αKG, which is an intermediate step in the tricarboxylic acid cycle [5, 6].

Several genes that influence hydroxymethylation are mutated in cancer. For example, mutations that disrupt *TET* expression or activity can affect the normal level of 5hmC. In addition, this level may be affected by changes in the expression of either genes coding for products that bind 5hmC or genes involved in demethylation [2].

Mutations that disrupt the functions of *TET* and *IDH1/2* genes cause changes in 5hmC levels of hematopoietic stem cells and have been shown to participate in the pathogenesis of hematopoietic malignancies [4, 7–9]. *TET2* mutations occur in 7–23% of patients with acute myeloid leukemia (AML) [4, 10–13] . The frequencies of *IDH1* and *IDH2* mutations in patients with AML are 5.5–14% and 8.7–19%, respectively [14]. A meta-analysis reported that *TET2* mutations negatively affect the prognosis of patients with normal karyotype (NK)-AML, while the prognostic implications of *IDH1/2* mutations in patients with NK-AML are unclear [14, 15]. However, the prognostic significance of *TET2* mutations in NK-AML is controversial [10, 13].

5hmC may function as an intermediate in demethylation and is known to be a transcriptional activator [16]. However, the prognostic implications of 5hmC have not been comprehensively evaluated in patients with NK-AML, especially in those affected by mutations. Additionally, the clinical significance of 5hmC levels has not been fully evaluated in patients with NK-AML. Herein, we evaluated the correlation of 5hmC levels with mutant alleles, as well as the significance of 5hmC levels in the context of survival and relapse risk.

## RESULTS

### 5hmC level and somatic mutations

The characteristics of these 375 patients are summarized in Table 1. The median 5hmC level was 0.065% (0.001–1.000). The levels of 5hmC deviated from the standard normal distribution and were therefore re-analyzed using a log scale.

**Table 1:** Patient characteristics according to the 5-hydroxymethylcytosine levels

	Total	Low 5hmC group	Intermediate 5hmC group	High 5hmC group	*P*-value^1)^
No. of patients (%)	375	124 (33.1)	125 (33.3)	126 (33.6)	NA
5hmC, % (range)	0.065 (0.001- 1.000)	0.030 (0.001- 0.051)	0.065 (0.051- 0.089)	0.093 (-3.05- 1.000)	NA
Age in years, median (range)	52 (15-83)	55 (20-83)	54 (15-83)	47 (15-84)	<0.001^2),3)^
Gender, male (%)	190 (50.7)	62 (50.0)	62 (49.6)	66 (51.7)	0.859
WBC, x 10^9^/L, median (range)	27.0 (0.3-397.2)	42.7 (0.7-333.2)	27.3 (0.5-397.2)	15.4 (0.3-292.5)	<0.001^2),3)^
Marrow blast, % (range)	72 (1-100)	79 (2-100)	70 (1-100)	70 (10-100)	<0.001^2),4)^
Achievement of CR, (%)	309/375 (82.4)	106 (85.5)	98 (78.4)	105 (83.3)	0.322
Received allogeneic SCT	106/375 (28.3)	31/124(25.0)	32/125 (25.6)	43/126 (34.1)	0.184
5-year relapse risk, % (95% CI)	44.7% (38.8-50.5)	39.7 (30.0-49.2)	43.5 (33.0-53.4)	48.9 (39.9-58.2)	0.593
5-year EFS rate, % (95% CI)	33.0% (27.9-38.1)	34.1 (25.1-43.1)	31.9 (23.3-40.5)	30.4 (22.2-38.6)	0.835
5-year OS rate, % (95% CI)	37.4% (32.1-42.7)	40.3 (31.1-49.5)	38.0 (29.2-46.8)	31.4 (23.0-39.8)	0.760
*IDH1/2* mutated, %	77/375 (20.5)	42 (33.9)	33 (26.4)	2 (1.6)	<0.001
*TET2* mutated, %	49/375 (13.1)	23 (18.5)	23 (18.4)	3 (2.4)	<0.001
*FLT3*-ITD mutated, %	104/375 (27.7)	35 (28.3)	35 (28.0)	34 (27.0)	0.973
*NPM1* mutated, %	167/375 (44.5)	62 (50.0)	60 (48.0)	45 (35.7)	0.048
*CEBPA* double mutated, %	48/374 (12.8)	11 (8.9)	13 (10.4)	24/125 (19.2)	0.031
*DNMT3A* mutated, %	124/374 (33.2)	48 (38.7)	46 (36.8)	30/125 (24.0)	0.027
*WT1* mutated, %	34/374 (9.1)	9 (7.3)	8 (6.4)	17/125 (13.6)	0.097
*NRAS* mutated, %	42/374 (11.2)	11 (8.9)	16 (12.8)	15/125 (12.0)	0.584
*FAT1* mutated, %	23/374 (6.1)	5 (4.0)	7 (5.6)	11/125 (8.8)	0.279
*ASXL1* mutated, %	26/369 (7.0)	15 (12.4)	5/124 (4.0)	6/124 (4.8)	0.019
*DNAH11* mutated, %	16/374 (4.3)	3 (2.4)	6 (4.8)	7/125 (5.6)	0.435
*GATA2* mutated, %	12/363 (3.3)	2/119 (1.7)	3/121 (2.5)	7/123 (5.7)	0.180

^1)^ The *p*-values refer to comparisons among three groups according to levels of *5hmC*.

^2)^ The *p*-values refer to comparisons between groups with low vs. high 5hmC levels.

^3)^ The *p*-values refer to comparisons between groups with intermediate vs. high 5hmC levels.

^4)^ The *p*-values refer to comparisons between groups with low vs. intermediate 5hmC levels.

Abbreviations: 5hmC, 5-hydroxymethylcytosine; WBC, white blood cells; CR, complete remission; SCT, stem cell transplantation; EFS, event-free survival; CI, confidence interval; OS, overall survival

The prevalence rates of mutations were 13.1% (n=49/375) for *TET2^mut^*, 6.7% (n=25/375) for *IDH1^mut^*, and 13.9% (52/375) for *IDH2^mut^*. The mutation rate of *TET2* or *IDH1/2* was 33.1% (n=124/375). Of the 375 patients analyzed, 59 different *TET2* mutations were detected in 49 of these patients (13.1%). Of these 59 *TET2* mutations, 13 were nonsense, 28 were frameshift, and 18 were missense. *TET2* gene double mutations were detected in 12 patients, while a homozygous *TET2* mutation was observed in 14 patients. The position and type of *TET2* mutations are described in Supplementary Figure 1. Twenty-five patients had an *IDH1* mutation in codon 132, whereas 52 patients had an *IDH2* mutation in codon 140 (n = 45) or codon 172 (n = 7).

The prevalence rates of other mutations were: *FLT3*-ITD^pos^, 27.7% (104/375); *NPM1*^mut^, 44.5% (167/375); *DNMT3A*^mut^, 33.2% (124/374); *NRAS*^mut^, 11.2% (42/374); *CEBPA*^mut^ (double), 12.8% (48/374); *WT1*^mut^, 9.1% (34/374); *ASXL1*^mut^, 7.0% (26/369); *FAT1*^mut^, 6.1% (23/374); *DNAH11*^mut^, 4.3% (16/374); and *GATA2*^mut^, 3.3% (12/363) (Table 1).

### 5hmC level inversely correlates with adverse clinical factors of treatment outcomes

We analyzed the levels of 5hmC according to the clinical factors with adverse outcomes. Older age (r= −0.151, *p*=0.027), high WBC count (*r*= −0.195, *p*<0.001), high blast percentage in bone marrow (*r*= −0.129, *p*=0.013), and high peripheral blast counts (*r*= −0.183, *p*<0.001) were inversely correlated with a low 5hmC level on a log scale. The log(5hmC) levels were not significantly correlated with gender (p=0.372) or CR achievement (*p*=0.807). In the sub-classification of the 5hmC values into tertiles, the high 5hmC group was younger and had a lower WBC count than the low or intermediate group. The low 5hmC group had a higher bone marrow blast percent than the intermediate or high 5hmC group (Table 1). *NPM1*^mut^, *DNMT3A*^mut^ and *ASXL1*^mut^ were observed more frequently in the low 5hmC groups, while *CEBPA*^dm^ was more frequent in the high 5hmC group (*p* <0.05) (Table 1).

### 5hmC levels correlate with the presence of *TET2* or *IDH1/2* mutations

**Figure 1:**
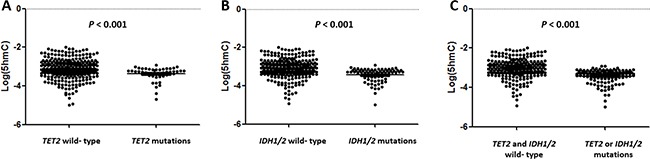
5-Hydroxymethycytosine (5hmC) levels are decreased in patients with *TET2* and *IDH* mutations Patients with *TET2* **A.** *IDH1/2* **B.** and *TET2* or *IDH1/2* **C.** mutations had significantly lower 5hmC levels than those with the *TET2* wild type, *IDH1/2* wild type, or both wild types (all P<0.001).

We examined whether 5hmC values correlated with *TET2* or *IDH1/2*mutation status. The log(5hmC) levels were lower in *TET2* or *IDH1/2* mutated groups than in the wild type (Figure 1). The levels of 5hmC were as follows: *TET2*^mut^ (median 0.051%, range 0.002–0.120), *TET2*^wild^ (median 0.070%, range 0.001–0.999), *IDH1*^mut^ (median 0.044%, range 0.004–0.073), *IDH1*^wild^ (median 0.069%, range 0.001–0.999), *IDH2*^mut^ (median 0.050%, range 0.001–0.118), *IDH2*^wild^ (median 0.069, range 0.001–0.999), any mutation of *TET2* or *IDH1/2* (median 0.048%, range 0.001–0.120), and *TET2*^wild^ and *IDH1/2*^wild^ (median 0.086, range 0.001–0.999). *TET2*^mut^, *IDH1*^mut^, and *TET2* or *IDH1*/*2* mutated patients had significantly lower levels of log(5hmC) than patients without mutations (all *p*<0.001) (Figure 1, Table 2). Older age, a high WBC count and a higher blast percentage in bone marrow were observed in the *TET2*^mut^ or *IDH1/2*^mut^ group and each mutated group. Low rate of undergoing allogeneic stem cell transplantation (SCT) at the first complete remission (CR1) was observed in the *TET2*^mut^ or *IDH1/2*^mut^ group and *TET2*^mut^ group (Table 2).

**Table 2:** Patient characteristics and levels of 5-hydroxymethylcytosine according to *TET2/IDH* mutation status

	Total	*TET2*^wild^ and *IDH1/2* ^wild^	*TET2*^mut^ or *IDH1/2*^mut^	*P*-value^1)^	*TET2*^mut^	*TET2*^wild^	*P*-value^2)^	*IDH1/2*^mut^	*IDH1/2* ^wild^	*P*-value^3)^
No. of patients (%)	375	251 (66.9)	124 (33.1)	NA	49 (13.1)	326 (86.9)	NA	77 (20.5)	298 (79.5)	NA
Age, years, median (range)	52 (15-83)	50 (15-84)	61 (16-83)	<0.001	63 (16-83)	50 (15-84)	<0.001	54 (16-75)	51 (15-84)	<0.001
Gender, male	190 (50.7)	125 (50.0)	65 (52.4)	0.562	23 (46.9)	167 (51.2)	0.562	42 (54.5)	148 (49.8)	0.461
WBC, x 10^9^/L , median (range)	27.0 (0.3-397.2)	24.9 (0.3-397.2)	36.7 (0.5-333.2)	<0.001	41.7 (0.9-282.0)	25.1 (0.3-397.2)	<0.001	25.2 (0.5-333.2)	27.3 (0.3-397.2)	<0.001
Marrow blast, % (range)	72 (1-100)	70 (1-100)	79 (2-100)	<0.001	75 (3-100)	72 (1-100)	<0.001	80 (2-100)	69 (1-100)	<0.001
5hmC, % (range)	0.065 (0.001- 0.99)	0.086 (0.001-1.000)	0.048 (0.001-0.120)	<0.001	0.051 (0.002- 0.120)	0.070 (0.001-0.999)	<0.001	0.047 (0.001 -0.094)	0.074 (0.001- 1.00)	<0.001
CR achievement, (%)	309/375 (82.4)	212 (84.8)	97 (78.2)	0.136	38 (77.6)	271 (83.1)	0.339	60 (77.9)	249 (83.6)	0.247
Received allogeneic SCT in CR1	106/309 (28.3)	81/212 (38.2)	25/97 (25.8)	0.033	7/38 (18.4)	99/271 (36.5)	0.028	20/60 (33.3)	86/249 (35.0)	0.860
5-year relapse risk, % (95% CI)	44.7 (38.8-50.5)	43.2 (36.1-50.0)	48.3 (37.1-58.6)	0.484	49.3 (30.9-65.4)	44.1 (37.8-50.2)	1.00	46.2 (32.5-58.9)	44.2 (37.6-50.6)	0.708
5-year EFS, % (95% CI)	33.0 (27.9-38.1)	34.8 (28.5-41.1)	29.2 (20.4-38.0)	0.260	26.3 (13.0-39.6)	34.0 (28.5-39.5)	0.334	30.7 (26.0-53.0)	33.6 (27.9-39.3)	0.450
5-year OS, % (95% CI)	37.4 (32.1-42.7)	39.0 (32.5-45.5)	34.1 (25.1-43.1)	0.303	34.6 (20.1-49.1)	37.9 (32.2-43.6)	0.502	33.3 (21.9-44.7)	38.5 (32.6-44.4)	0.358

^1)^ The *p*-values refer to comparisons between groups with *TET2*^wild^ and *IDH1/2*^wild^ vs. *TET2*^mut^ or *IDH1/2*^mut^.

^2)^ The *p*-values refer to comparisons between groups with *TET2*^wild^ vs. *TET2*^mut^.

^3)^ The *p*-values refer to comparisons between groups with *IDH1/2*^wild^ vs. *IDH1/2*^mut^.

Abbreviations: 5hmC, 5-hydroxymethylcytosine; CR1, first complete remission; SCT, stem cell transplantation; RI, relapse incidence; CI, confidence interval; EFS, event-free survival; OS, overall survival; RI, relapse incidence; WBC, white blood cells

### 5hmC levels are not prognostic with respect to the risk of relapse, EFS, or OS

Of 375 patients receiving induction chemotherapy, 309 (82.4%) achieved complete remission (CR). Of the 309 patients who achieved CR, 106 patients received allogeneic SCT as consolidation therapy. At a median follow-up of 55.0 (range 0.9–179.8) months among survivors, 66 patients (17.6%) failed to achieve CR, 129 patients (34.4%) relapsed after CR was achieved, and 158 patients (42.1%) died either from relapse (n=109, 39.1%) or from other causes (n=49, 13.1%). The relapse risk at 5 years was 44.7% (95% CI 38.8–50.5%). The event-free survival (EFS) and overall survival (OS) rates at 5 years were 33.0% (95% CI 27.9–38.1%) and 37.4% (95% CI 32.1–42.7%), respectively.

**Figure 2:**
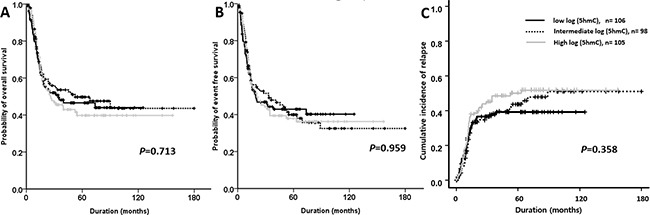
Outcomes of patients with normal karyotype acute myeloid leukemia according to the 5-hydroxymethylcytosine (5hmC) levels Overall survival **A.** event-free survival **B.** and relapse incidence **C.** are shown.

At low, intermediate, and high levels of 5hmC, the CR rates were 85.5%, 78.4%, and 83.3%, respectively (*p*=0.322). The relapse risk, EFS, and OS did not differ significantly among patients in the different categories. Specifically, for the groups with low, intermediate, and high levels of 5hmC, the relapse risk rates at 5 years were 39.7%, 43.5%, and 48.9% (p=0.593), the EFS rates at 5 years were 34.1%, 31.9%, and 30.4% (p=0.835), and the OS rates at 5 years were 40.3%, 38.0%, and 31.4%, respectively (p=0.760) (Figure 2). There were no clinically significant differences in relapse risk, OS, and EFS according to the levels of 5hmC (all *p*>0.05).

Univariate analysis showed that *NPM1*^mut^ (*p*=0.006), *CEBPA*^dm^ (*p*=0.002), and receiving allogeneic SCT (*p*=0.003) were associated with longer OS, while age (≥65 years) (*p*=0.020), *DNMT3A*^mut^ (*p*=0.011), and *FLT3*-ITD^pos^ (*p*=0.001) were associated with worse OS. Furthermore, age (≥65 years) (*p*=0.002), WBC count (*p*=0.034), peripheral blast count (*p*=0.045), *FLT3-*ITD^pos^ (*p*=0.008), and *ASXL1*^mut^ (*p*=0.002) were associated with worse outcomes for EFS, while *NPM1*^mut^ (*p*=0.003), *CEBPA*^dm^ (*p*=0.001), and receiving allogeneic SCT (*p*=0.003) were associated with favorable outcomes for EFS. *CEBPA*^dm^ (*p*=0.003) and receiving allogeneic SCT (*p*<0.001) were associated with a lower relapse risk, while age (≥65 years) (*p*=0.003) and *FLT3-ITD*^pos^ (*p*<0.001) were associated with a higher relapse risk.

The results of multivariate analysis are shown in Table 3. The results of *NPM1*^mut^ and receiving allogeneic SCT were favorable, while *FLT3*-ITD^pos^ and *DNMT3A*^mut^ were poor risk factors for OS. The results of *NPM1*^mut^, *CEBPA*^dm^, and receiving allogeneic SCT were favorable, while *FLT3-*ITD^pos^ and *DNMT3A*^mut^ were poor risk factors, for EFS. In addition, age (≥65 years), *FLT3*-ITD^pos^, and *ASXL1*^mut^ were significantly associated with a higher relapse risk, while *NPM1*^mut^, *CEBPA*^dm^, and receiving allogeneic SCT reduced the relapse risk. There were no clinically significant differences in OS, EFS, and relapse risk according to the levels of 5hmC in multivariate analysis (all *p*>0.05).

**Table 3:** Univariate and multivariate analyses of overall survival (OS), event-free survival (EFS), and relapse risk in patients with normal karyotype acute myeloid leukemia and risk factors including genetic and clinical factors at diagnosis

Parameter	Variable	Univariate		Multivariate	
		HR	*p*-value	HR	*p-*value
OS	Age ≥65 years	1.480	0.020	1.240	0.339
	WBC count (cont)	1.000	0.110	1.000	0.759
	Peripheral blast count (cont)	1.000	0.129	1.000	0.605
	Allo SCT at CR1	0.593	0.003	0.552	0.002
	*NPM1* mutation	0.683	0.006	0.479	<0.001
	*CEBPA* double mutation	0.482	0.002	0.632	0.096
	*FLT3*-ITD positive	1.612	0.001	2.265	<0.001
	*DNMT3A* mutation	1.424	0.011	1.750	0.003
	*ASXL1* mutation	1.430	0.137	0.994	0.986
	5hmC (low)^1)^	1.000	0.869	0.983	0.924
	(intermediate)^2)^	1.009	0.954	0.852	0.362
	(high)^3)^	1.075	0.869	1.152	0.406
EFS	Age ≥65 years	1.656	0.002	1.274	0.248
	WBC count (cont)	1.000	0.034	1.000	0.876
	Peripheral blast count (cont)	1.000	0.045	1.000	0.777
	Allo SCT at CR1	0.593	0.003	0.414	<0.001
	*NPM1* mutation	0.679	0.003	0.459	<0.001
	*CEBPA* double mutation	0.462	0.001	0.576	0.033
	*FLT3*-ITD positive	1.454	0.008	2.087	<0.001
	*DNMT3A* mutation	1.266	0.083	1.662	0.005
	*ASXL1* mutation	1.982	0.002	1.549	0.163
	5hmC (low)^1)^	1.000	0.729	1.058	0.733
	(intermediate)^2)^	1.022	0.888	0.942	0.715
	(high)^3)^	1.057	0.729	1.123	0.476
Relapse risk	Age ≥65 years	1.944	0.003	1.5800	0.044
	WBC count	1.000	0.270	1.000	0.530
	Peripheral blast count	1.000	0.290	1.000	0.300
	Allo SCT at CR1	0.346	<0.001	0.347	<0.001
	*NPM1* mutation	0.741	0.092	0.500	<0.001
	*CEBPA* double mutation	0.372	0.003	0.373	0.005
	*FLT3*-ITD positive	1.981	<0.001	2.219	<0.001
	*DNMT3A* mutation	1.184	0.380	1.461	0.088
	*ASXL1* mutation	1.882	0.062	1.774	0.009
	5hmC (low)^1)^	0.797	0.250	0.803	0.171
	(intermediate)^2)^	0.951	0.780	0.916	0.650
	(high)^3)^	1.312	0.140	1.377	0.121

Abbreviations: WBC, white blood cells; 5hmC, 5-hydroxymethylcytosine; Allo SCT, allogeneic stem cell transplantation; CR1, first complete remission; HR, hazard ratio; cont, continuous variable

^1)^ The HR and *p*-values refer to comparisons between groups with low 5hmC levels vs. intermediate and high levels.

^2)^ The HR and *p*-values refer to comparisons between groups with intermediate 5hmC levels vs. low and high levels.

^3)^ The HR and *p*-values refer to comparisons between groups with high 5hmC levels vs. low and intermediate levels.

We sub-analyzed the significance of levels of 5hmC according to each clinically significant mutational status. The levels of 5hmC did not influence the survival or relapse risk in each mutational status, even including that of *TET2* or *IDH1/2* (Supplementary Figure 2 and Supplementary Table 3).

## DISCUSSION

We examined the prognostic implications and clinical significance of changes to 5hmC levels as a result of *TET2* or *IDH1/2* mutations. The log(5hmC) levels were found to be inversely correlated with age, white blood cell (WBC) count, and the percentage of blasts in bone marrow. Patients with *TET2* or *IDH1*/*2* mutations had significantly lower levels of log(5hmC) than patients without any *TET2* or *IDH1/2* mutations. However, when we sub-categorized the levels of 5hmC into tertiles, we found that low, intermediate, or high levels did not influence the achievement of CR. Furthermore, relapse risk, EFS, and OS were not found to be significantly different among the patients of any sub-group.

*TET2* and *IDH1/2* genes are important for regulating DNA methylation. Disruptions to their normal functions (i.e. alterations to DNA methylation) have been observed in several cancers. For example, in another study, it was demonstrated that AML patients with *TET2* or *IDH 1/2* mutations displayed decreased levels of 5hmC [12, 17–19]. The altered patterns of covalent cytosine modifications point to the potential for novel diagnostic, prognostic, and therapeutic applications. The level of 2-hydroxyglutarate in leukemic cells of AML patients (at the time of diagnosis) is suggested as an excellent surrogate marker for *IDH1/2* mutations [16]. *TET2* haploinsufficiency may contribute to abnormal myeloid transformation [7]. *TET2* mutation occurs early in leukemogenesis, suggesting its significance in the onset and progression of hematologic malignancies [20]. Given that the levels of 5hmC indicate the presence of *TET2* or *IDH1/2* mutations, we speculate that 5hmC could also be a predictive marker for the prognosis and detection of *TET2* or *IDH1/2* mutations.

The significance of 5hmC levels in AML is unclear. Kroeze et al. previously attempted to demonstrate the effect of 5hmC levels on AML [19]. Specifically, they showed that the levels of 5hmC during CR were normalized to those levels seen in healthy bone marrow and peripheral blood. This indicated that aberrant levels of 5hmC at diagnosis were an intrinsic property of leukemic cells [19]. Interestingly, they also demonstrated that high levels of 5hmC were associated with a poor prognosis (as per a multivariate analysis), along with variable results of the effects of *TET2* or *IDH1/2* mutations on survival. Since the group with high levels of 5hmC was small (8.2%, 17/206), the results cannot be generalized to all AML patients. In our cohort, there was no significant difference in survival according to the levels of 5hmC. We tried to sub-classify the 5hmC value using R partitioning to find the valuable cut-off range but, we did not find a significant cut-off value for levels of 5hmC in OS, EFS, and relapse risk. Therefore, we sub-classified the patients into low, intermediate, and high level tertile groups of 5hmC to determine clinical significance. The difference between our findings and those of previous studies can likely be explained by the higher proportion of patients >60 years of age (32.3%); furthermore, the effects of cytogenetic abnormalities on the levels of 5hmC were not considered in our study. The levels of 5hmC decline with aging, so the inclusion of older patients could influence the results of 5hmC [21]. We observed similar results in the NK-AML population with respect to the inverse correlation between log(5hmC) levels and age. WBC count and the percentage of blasts in bone marrow were also negatively correlated with log(5hmC) levels. In our previous study, *TET2* mutations were associated with older age and a high WBC count. This suggests that patients with high WBC counts have more *TET2* mutations, which might explain the correlation [13]. Our study shows that the 5hmC levels positively correlated with *CEBPA*^dm^, but negatively correlated with *NPM1*^mut^, *DNMT3A*^mut^ and *ASXL1*^mut^. To our knowledge, no report has directly explained the relationship of 5hmC levels and *CEBPA*^dm^, *NPM1*^mut^, *DNMT3A*^mut^, and *ASXL1*^mut^. Previously, we showed that *TET2*^mut^ is mutually exclusive with *CEBPA*^dm^, but has a positive correlation with *NPM1*^mut^ [13]. *DNMT3A*^mut^ and *CEBPA*^dm^ are also mutually exclusive, whereas *DNMT3A*^mut^ was positively correlated with *NPM1*^mut^ [22]. We speculate that mutations of epigenetic modifying genes influence the associated mutational status of the 5hmC groups.

Our results have significant clinical relevance because the study population was restricted, exclusively, to patients with NK-AML. Furthermore, all patients received induction chemotherapy, while any untreated patients were excluded. However, this study had a methodological limitation given that Sanger's fluorescent dideoxynucleotide chain termination sequencing analysis has a detection sensitivity of approximately 10% of mutant alleles. Recently, a novel technology for next-generation sequencing was developed. This technology recognizes unique sequences, provides depth of coverage and accuracy of sequencing [23], and offers a powerful tool that may become integral to resolving correlation clonal dynamics with levels of 5hmC. In this study, we did not demonstrate the prognostic significance of 5hmC levels. However, *TET2* mutations have been shown to predict the response of patients with myelodysplastic syndrome to hypomethylating agents [24]. Hypomethylating agents are used in the standard treatment of elderly patients with AML who are not eligible for standard induction therapy [25, 26]. The investigation of 5hmC levels in such a population could be a good prognostic marker for prediction of the response to hypomethylating agents and to tailor therapies and assess responses to anticancer drugs [12].

In summary, *TET2* or *IDH1/2* mutated patients had lower levels of 5hmC. Apart from affecting the methylation status of DNA, other processes may be influenced by altered levels of 5hmC in patients with NK-AML and *TET2*^wild^ and *IDH1/2*^wild^. 5hmC may not have prognostic value for predicting survival or relapse risk in patients with NK-AML who have been treated with intensive induction therapy.

## PATIENTS AND METHODS

### Patients and treatment

This study included patients diagnosed with NK-AML at seven participating institutions between October 1998 and September 2012. Out of 407 patients screened for this study, 375 DNA samples were available to evaluate the levels of 5hmC [13]. The median patient age was 52 (range 15–83) years and the subjects included 190 males (Supplementary Table 1). Most cases were AML not otherwise specified (88.8%), with some therapy-relapsed AML (2.9%) or AML with myelodysplasia-related changes (8.3%). Patients had received induction chemotherapy using a standard protocol [a 3-day course of anthracycline with a simultaneous 7-day course of cytosine arabinoside (Ara-C) or N^4^-behenoyl-1-b-d-arabinofuranosylcytosine (BHAC)] [13]. Idarubicin was administered daily at a dose of 12 mg/m^2^ or daunorubicin was administered at a dose of 60 mg/m^2^ on three consecutive days. Ara-C was administered daily at a dose of 100 mg/m^2^ and BHAC at a dose of 300 mg/m^2^ on seven consecutive days. In all, 210 patients were treated with idarubicin + Ara-C, 71 patients were treated with idarubicin + BHAC, and 94 patients received daunorubicin + Ara-C induction chemotherapy. Of 375 patients, 260 (69.3%) achieved complete remission (CR) after first induction chemotherapy. Seventy-Two patients received second induction chemotherapy (44 patients with first induction regimen, 13 patients with mitoxantrone based induction and, 15 patients with fludarabine based induction) and 43 patients achieved CR after second induction chemotherapy. Six out of 17 patients achieved CR after third induction chemotherapy. Patients who achieved CR received consolidation chemotherapy with or without allogeneic SCT, depending on the availability of an HLA-matched donor (related or unrelated). Genetic factors such as *FLT3*-ITD or *NPM1* mutation were not considered when deciding whether to perform allogeneic SCT for consolidative treatment. Written informed consent was obtained from all subjects for the genetic analysis of samples taken at the time of diagnosis. The study was approved by the Institutional Review Board of Chonnam National University Hwasun Hospital, South Korea.

### Gene mutation analyses

Cryopreserved bone marrow or peripheral blood samples taken at diagnosis were archived. Genomic DNA was extracted using QIAamp DNA blood mini-kits (QIAGEN, Valencia, CA, USA), as per the manufacturer's protocol. Mutation analyses were performed using Sanger sequencing and polymerase chain reaction (PCR). *TET2*, *FLT3*-ITD, and *NPM1* mutation testing was performed as described previously [27, 28]. *TET2* missense mutations were included in the analysis only when they were located within one of two evolutionarily conserved domains (amino acids 1,104–1,478 or 1,845–2,002) and identical *TET2* mutations in both alleles were defined as homozygous [29, 30]. The *DNMT3A*, *WT1*, *NRAS*, *ASXL2*, and *IDH1/2* mutation analysis was performed as reported previously [31, 32] [29–35]. The *CEBPA, FAT1*, *DNAH11*, and *GATA2* were amplified by genomic PCR; overlapping PCR products covering the entire coding sequence were generated and sequenced using the PCR primers in Supplementary Table 2. Amplification featured initial denaturation at 95°C for 5 min, followed by 40 cycles of 94°C for 30 s, 62°C for 30 s, and 72°C for 1 min, and a final 10-min extension at 72°C. The amplification products were sequenced on an ABI 3100 platform using a cycle sequencing kit (BigDye Terminator; Applied Biosystems, Foster City, CA, USA).

### Level of 5hmC

The 5hmC levels were measured in 375 DNA samples using Quest 5hmC^™^ DNA ELISA Kits (Zymo Research, Boston, MA, USA). DNA (100 ng) from each patient or control was used for quantification. Biochemical assays were performed according to the manufacturer's recommendations and samples were read in a spectrophotometer at 450 nm. Absolute quantification was performed using the concentrations of positive control samples by the standard curve method. The amount of 5hmC was calculated from 100-ng samples of single-stranded DNA and is reported as a percentage (%).

### Response and survival endpoints

The definition of CR followed reported criteria [36]. Relapse risk was defined as the time from achieving remission to the date of relapse in all patients who achieved CR, considering the competing risk of death without relapse. Non-relapse mortality (NRM) was defined as death occurring in the absence of relapse. EFS was defined as the time from commencing induction chemotherapy to the date of death from any cause, relapse, or non-achievement of CR, whichever occurred first. OS was defined as the time from the start of induction chemotherapy to the date of last follow-up, or death from any cause. Patients undergoing allogeneic HSCT were not censored at the time of transplantation.

### Statistical analyses

First, we tried to sub-classify the 5hmC value using R partitioning to find the valuable cut-off range. However, we could not find a significant cut-off value for 5hmC (*p*>0.05). Therefore, we simply sub-classified the 5hmC values into tertiles. Descriptive statistics are presented as frequencies (%) and medians (with ranges) for categorical and continuous variables, respectively. The clinical characteristics and treatment outcomes of each mutation were compared with each other. The χ^2^ test was used to compare the differences in categorical data distributions, while the Wilcoxon rank-sum test was used to evaluate the differences between continuous variables. ANOVA was used to evaluate the differences between continuous variables and compare three groups for statistical significance. Pearson and Spearman correlation coefficients were used to calculate the correlations between two continuous variables. EFS and OS were calculated using the Kaplan–Meier method; the differences among groups were compared using the log-rank test and Cox's proportional hazard model for univariate and multivariate analyses, respectively. Age, WBC count, peripheral blast count, receiving allogeneic SCT, frequencies of *NPM1*^mut^, *CEBPA*^mut^ (double), *FLT3*-ITD^pos^, *DNMT3A*^mut^, and *ASXL1*^mut^, as well as the 5hmC level were included in the final multivariate model. Covariates with parameters that were significant in univariate analyses were included in the multivariate analysis. We also included the 5hmC level in the multivariate analysis to identify its clinical significance. The incidences of relapse and NRM were calculated using a cumulative incidence method that considered competing risks. Gray's test and the Fine–Gray test were used for univariate and multivariate comparisons, respectively [37]. *P*-values < 0.05 were considered to be significant. Hazard ratios (HRs) and 95% confidence intervals (CIs) were estimated using a predetermined value for reference risk unity. All statistical analyses were performed using SPSS ver. 21.0 (SPSS, Chicago, IL, USA) and EZR software in ‘R’ language (available at http://www.jichi.ac.jp/saitama-sct/SaitamaHP.files/statmedEN.html) [37].

## SUPPLEMENTARY MATERIALS FIGURES AND TABLES




