# Supplementary material for: 5-Hydroxymethylcytosine correlates with epigenetic regulatory mutations, but may not have prognostic value in predicting survival in normal karyotype acute myeloid leukemia
Source: Oncotarget. 2016 Dec 26;8(5):8305–14. doi: 10.18632/oncotarget.14171 (PMC5352402; doi:10.18632/oncotarget.14171)
Supplement: Supplementary file 1 [file oncotarget-08-8305-s001.pdf]

5-Hydroxymethylcytosine correlates with epigenetic regulatory mutations, but may not have prognostic value in predicting survival in normal karyotype acute myeloid leukemia

SUPPLEMENTARY FIGURES AND TABLES

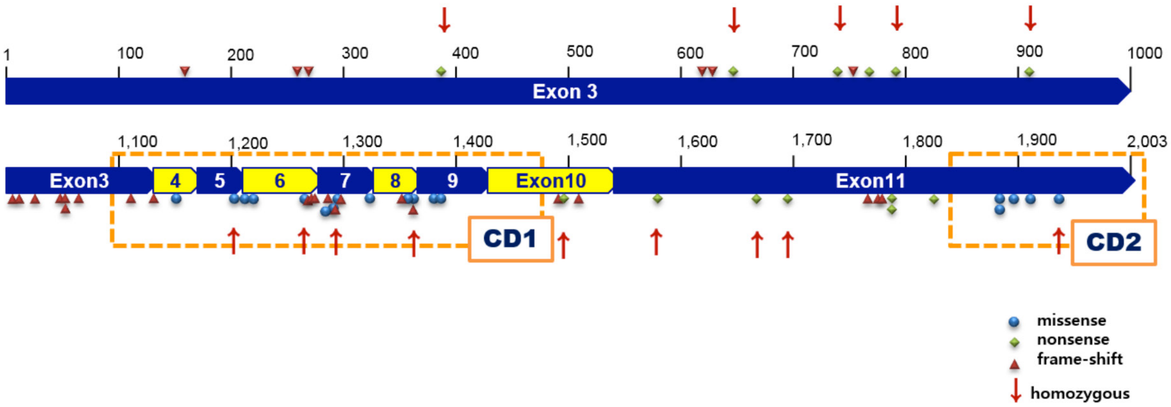

Supplementary Figure 1: Position and type of *TET2* mutations.

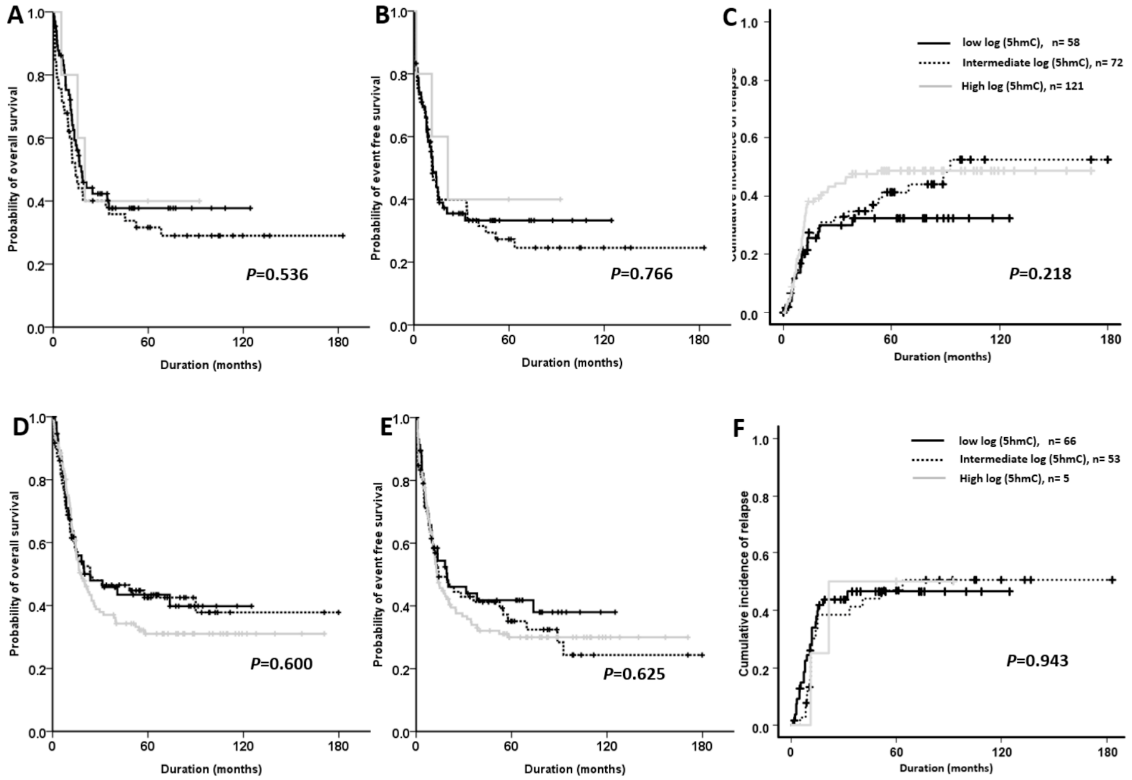

Supplementary Figure 2: Outcomes of patients with normal karyotype acute myeloid leukemia according to 5-hydroxymethylcytosine (5hmC) levels *in TET2* or *IDH1/2* mutated groups A, B, C. and both wild types **D, E, F.** Overall survival (A, D), event-free survival (B, E), and relapse incidence (C, F) are shown.

Supplementary Table 1: Clinical characteristics of 375 patients with acute myeloid leukemia of normal karyotype

**Parameter; no. of patients (%)**	**Total**	**(%)**
Number of patients	375	100
Age in years, median (range)	52 (15–83)	
Gender, male	190	50.7
WBC, median value, × 10^9/L (median)	27.0 (0.3–397.2)	
Bone marrow blasts, %, median (range)	72 (1–100)	
WHO classification		
AML with myelodysplasia-related changes	31	8.3
Therapy related myeloid neoplasms	11	2.9
Acute myeloid leukemia, NOS		
AML with minimal differentiation	16	4.3
AML without maturation	58	15.4
AML with maturation	129	34.4
Acute myelomonocytic leukemia	58	15.4
Acute monocytic leukemia	42	11.2
Acute erythroid leukemia	19	5.1
Acute megakaryocytic leukemia	7	1.9
Acute panmyelosis with myelofibrosis	4	1.1
Induction therapy		
Idarubicin + Ara-C	210	56.0
Idarubicin + BHAC	71	18.9
Daunorubicin + Ara-C	94	25.1
Allogeneic stem cell transplantation at first CR	106	28.3

Abbreviations: WBC, white blood cells; WHO, World Health Organization; AML, acute myeloid leukemia; NOS, not otherwise specified; Ara-C, cytosine arabinoside; BHAC, N^4-behenoyl-1-b-D-arabinofuranosyl cytosine; CR, complete remission.

Supplementary Table 2: The sequences of PCR primers used for analysis of *CEBPA*, *FAT1*, *DNAH11* and *GATA2* mutational status

Gene	Amplicon	F-Primer (5'-3')	R-Primer (5'-3')
CEBPA	Fragment 1	CACCTGCAGTTCCAGATCG	AGGCCAGGCTTTCAGGAG
	Fragment 2	GCCGGGAGAACTCTAACTCC	GCTTGGCTTCATCCTCCTC
	Fragment 3	GCTGGTGATCAAGCAGGAG	GGTCATTGTCACTGGTCAGC
FAT1	Fragment 1	ACCAGAAGGGCAGCAGAC	GGTAATGGTTTCAGGGGTTG
	Fragment 2	TTTTGTGTGCTATCAGCTTG	TTTTGGGAACTTGAGTAATACA
	Fragment 3	ACCAGAATCCCCATCCGTA	CTCATGGGCATTTCGTGAC
	Fragment 4	CTGCTCCCATACAACTTCAT	ACATTGATAGTGAAAGCACTTG
	Fragment 5	TCCCCAAGGTAACGATTCTG	CGCGCCTCCACATTAGTATT
	Fragment 6	CCCACCTCATACAGCGTTTC	GGGCGGAGTTCCTTTATCTT
	Fragment 7	TGAAGGGACAATTCCCAGAG	TGGGGTATCCCAAGGTCATA
	Fragment 8	TCCACCCGAGTTTTTACAGG	CTGGCTTTCCCTTGTCTTTG
	Fragment 9	CCCTCAGATGGACAACCTGT	GTCTGGCTGTGCTGTGTGTT
	Fragment 10	CATGGAAGGCTGCAGTACAA	GCTCCTGCCCATTCAAATAA
DNAH11	Fragment 1	CGCCTCAGGAATTGTGAGTT	CTCCCAGGACATCAAAAGGA
	Fragment 2	TTTTCAGCCAGTGCACAAAG	AAATTTCACCAGCTGGCATC
	Fragment 3	AGATTTGCTGGGTGATTGCT	GCCGACTAAGTGTTGGTGGT
GATA2	Fragment 1	GAGGCAAGGCACCCCTCTT	GAGGTGGGCGTGGGAGTC
	Fragment 2	GGCAAAGCGTCTGCATTT	AACCGTGTGCCTGAGAGG

Supplementary Table 3: Clinical outcomes of normal karyotype acute myeloid leukemia patients according to mutational status and levels of 5-hydroxymethylcytosine (5hmC)

See Supplementary File 1
