# Supplementary material for: 5-Hydroxymethylcytosine correlates with epigenetic regulatory mutations, but may not have prognostic value in predicting survival in normal karyotype acute myeloid leukemia
Source: Oncotarget. 2016 Dec 26;8(5):8305–14. doi: 10.18632/oncotarget.14171 (PMC5352402; doi:10.18632/oncotarget.14171)
Supplement: Supplementary file 2 [file oncotarget-08-8305-s002.doc]

Supplementary Table 3. Clinical outcomes of normal karyotype acute myeloid leukemia patients according to mutational status and levels of 5-hydroxymethylcytosine (5hmC)

| Parameter | No. of patients (%) | 5-year OS, (95% CI) | *p* value | 5-year EFS (95% CI) | *p* value | 5-year RI (95% CI) | *p* value | |
| --- | --- | --- | --- | --- | --- | --- | --- | --- |
| **All patients** | 375 (100) | 37.4% (32.1-42.7) |  | 33.0% (27.9-38.1) |  | 44.7% (38.8-50.5) |  | |
| ***FLT3*-ITD negative** | 271 |  | 0.618 |  | 0.543 |  | 0.292 | |
| 5hmC levels (low) | 89 (32.8) | 48.1% (37.1-59.1) |  | 44.1% (33.3-54.9) |  | 31.3% (21.0-42.2) |  | |
| 5hmC levels (intermediate) | 90 (33.2) | 43.9% (33.5-54.3) |  | 34.3% (23.9-44.7) |  | 38.2% (26.0-50.2) |  | |
| 5hmC levels (high) | 92 (33.9) | 37.5% (26.9-48.1) |  | 34.4% (24.2-44.6) |  | 47.4% (34.6-59.1) |  | |
| ***FLT3*-ITD positive** | 104 |  | 0.526 |  | 0.526 |  | 0.965 | |
| 5hmC levels (low) | 35 (33.7) | 15.4% (0-31.9) |  | 14.2% (0-29.6) |  | 65.1% (36.8-83.2) |  | |
| 5hmC levels (intermediate) | 35 (33.7) | 26.4% (10.7-42.1) |  | 25.9% (10.4-41.4) |  | 56.8% (35.2-73.7) |  | |
| 5hmC levels (high) | 34 (32.7) | 23.2% (8.1-38.3) |  | 24.2% (9.1-39.3) |  | 63.0% (42.1-78.1) |  | |
| ***NPM1* wild** | 208 |  | 0.813 |  | 0.801 |  | 0.472 | |
| 5hmC levels (low) | 62 (29.8) | 34.1% (21.6-46.6) |  | 32.3% (19.8-44.8) |  | 45.0% (30.0-58.9) |  | |
| 5hmC levels (intermediate) | 65 (31.3) | 30.8% (19.0-42.6) |  | 25.7% (14.1-37.3) |  | 42.6% (26.8-57.6) |  |  |
| 5hmC levels (high) | 81 (38.9) | 25.0% (14.4-35.5) |  | 23.1% (13.3-32.9) |  | 58.3% (43.9-70.1) |  |  |
| ***NPM1* mutated** | 167 |  | 0.926 |  | 0.648 |  | 0.649 |  |
| 5hmC levels (low) | 62 (37.1) | 46.6% (33.3-59.9) |  | 41.5% (28.6-54.4) |  | 33.8% (21.3-46.7) |  |  |
| 5hmC levels (intermediate) | 60 (35.9) | 48.0% (35.1-60.9) |  | 38.6% (25.7-51.5) |  | 44.4% (29.9-57.9) |  |  |
| 5hmC levels (high) | 45 (26.9) | 47.9% (32.6-63.2) |  | 46.9% (31.8-62.0) |  | 41.6% (25.7-56.8) |  |  |
| ***CEBPA* wild/single mutated** | 326 |  | 0.761 |  | 0.780 |  | 0.164 |  |
| 5hmC levels (low) | 113 (34.7) | 35.7% (26.1-45.3) |  | 32.0% (22.8-41.2) |  | 42.9 % (32.3-53.2) |  |  |
| 5hmC levels (intermediate) | 112 (34.4) | 37.8% (28.4-47.2) |  | 30.7% (21.5-39.9) |  | 45.9% (34.4-56.7) |  |  |
| 5hmC levels (high) | 101 (31.0) | 27.2% (17.8-36.6) |  | 25.5% (16.7-34.3) |  | 58.3% (46.3-68.6) |  |  |
| ***CEBPA* double mutated** | 48 |  | 0.490 |  | 0.357 |  | 0.542 |  |
| 5hmC levels (low) | 11 (22.9) | 81.8% (59.1-100) |  | 81.8% (59.1-100) |  | 9.1% (0.4-34.7) |  |  |
| 5hmC levels (intermediate) | 13 (27.1) | 51.9% (23.9-79.9) |  | 42.2% (13.4-71.0) |  | 26.2% (5.5-53.9) |  |  |
| 5hmC levels (high) | 24 (50.0) | 52.1% (27.4-76.8) |  | 54.8% (33.6-76.0) |  | 22.9% (10.7-58.3) |  |  |
| ***DNMT3A* wild** | 250 |  | 0.542 |  | 0.587 |  | 0.422 |  |
| 5hmC levels (low) | 76 (30.4) | 43.9% (32.1-55.7) |  | 39.0% (27.4-50.6) |  | 37.4% (25.2-49.6) |  |  |
| 5hmC levels (intermediate) | 79 (31.6) | 44.3% (32.7-55.9) |  | 33.5% (22.3-44.7) |  | 45.6% (32.2-57.9) |  |  |
| 5hmC levels (high) | 95 (38.0) | 34.0% (23.4-44.6) |  | 31.8% (21.8-41.8) |  | 47.5% (35.4-58.7) |  |  |
| ***DNMT3A* mutated** | 124 |  | 0.566 |  | 0.600 |  | 0.541 |  |
| 5hmC levels (low) | 48 (38.7) | 34.5% (19.8-49.2) |  | 33.9% (19.4-48.4) |  | 41.6% (25.6-56.8) |  |  |
| 5hmC levels (intermediate) | 46 (37.1) | 30.0% (16.3-43.7) |  | 28.2% (14.3-42.1) |  | 40.1% (22.2-57.4) |  |  |
| 5hmC levels (high) | 30 (24.2) | 28.7% (12.2-45.1) |  | 29.1% (12.6-45.6) |  | 57.8% (36.7-74.1) |  |  |
| ***ASXL1* wild** | 343 |  | 0.971 |  | 0.783 |  | 0.404 |  |
| 5hmC levels (low) | 106 (30.9) | 40.3% (30.3-50.3) |  | 38.5% (28.5-48.5) |  | 37.7% (27.4-47.9) |  |  |
| 5hmC levels (intermediate) | 119 (34.7) | 40.3% (31.1-49.5) |  | 32.6% (23.6-41.6) |  | 44.7% (33.8-55.0) |  |  |
| 5hmC levels (high) | 118 (34.4) | 35.3% (26.1-44.5) |  | 33.3% (24.5-42.1) |  | 47.7% (37.4-57.4) |  |  |
| ***ASXL1* mutated** | 26 |  | 0.710 |  | 0.968 |  | 0.439 |  |
| 5hmC levels (low) | 15 (57.7) | 35.7% (10.6-60.8) |  | 20.0% (0-40.2) |  | 60.0% (21.6-84.3) |  |  |
| 5hmC levels (intermediate) | 5 (19.2) | 20.0% (0-55.1) |  | 20.0% (0-55.1) |  | 0% |  |  |
| 5hmC levels (high) | 6 (23.1) | 0% |  | 0% |  | 100% |  |  |
| ***TET2* and *IDH1/2* wild** | 251 |  | 0.600 |  | 0.625 |  | 0.943 |  |
| 5hmC levels (low) | 58 (23.1) | 43.4% (29.9-56.9) |  | 41.8% (28.3-55.3) |  | 46.5% (32.1-59.7) |  |  |
| 5hmC levels (intermediate) | 72 (28.7) | 42.5% (30.5-54.5) |  | 35.1% (23.1-47.1) |  | 47.1% (29.9-62.5) |  |  |
| 5hmC levels (high) | 121 (48.2) | 31.0% (22.4-39.6) |  | 30.0% (21.6-38.4) |  | 50.0% (2.3-88.1) |  |  |
| ***TET2* or *IDH1/2* mutated** | 124 |  | 0.536 |  | 0.766 |  | 0.218 |  |
| 5hmC levels (low) | 66 (53.2) | 37.7% (25.2-50.2) |  | 33.3% (21.3-45.3) |  | 32.4% (19.7-45.7) |  |  |
| 5hmC levels (intermediate) | 53 (42.7) | 31.6% (18.7-44.5) |  | 27.3% (14.8-39.8) |  | 41.3% (28.0-54.2) |  |  |
| 5hmC levels (high) | 5 (4.0) | 40.0% (0-82.9) |  | 40.0% (0-82.9) |  | 48.9% (38.6-58.3) |  |  |

Abbreviations: OS, overall survival; EFS, event-free survival; RI, relapse incidence; NRM, non-relapse mortality
